# Construction of naringenin-loaded chitosan/sodium alginate nanocomposite gels and their controlled-release kinetics in simulated gastrointestinal media

**DOI:** 10.1039/d6ra05537k

**Published:** 2026-09-11

**Authors:** Shuai Yan, Jiuba Zhang, Lianlin Su, Yinzi Yue, Shuguang Zhen

**Affiliations:** a Department of Anorectal Surgery, Suzhou TCM Hospital Affiliated to Nanjing University of Chinese Medicine Suzhou 215009 China suz009@njucm.edu.cn; b Pratt School of Engineering, Duke University Durham NC 27708 USA; c School of Pharmacy, Nanjing University of Chinese Medicine Nanjing 210023 China

## Abstract

Naringenin has gastrointestinal pharmacological potential, but poor aqueous solubility, rapid crystallization and limited intestinal residence constrain oral formulation performance. This study evaluated whether coupled tripolyphosphate (TPP) and calcium-ion gelation could produce chitosan/sodium alginate (CS/ALG) nanocomposite gels that balance drug loading, gastric protection and intestinal release. Ten independently prepared formulations varied polymer composition, crosslinker density and naringenin feed. The optimized CA-21 formulation had a hydrodynamic diameter of 227.2 ± 9.4 nm, polydispersity index (PDI) of 0.14 ± 0.02, zeta potential of 18.9 ± 4.5 mV, encapsulation efficiency (EE) of 90.5 ± 4.7%, drug loading (DL) of 10.0 ± 1.5% and dried-product yield of 89.3 ± 2.2%. Transmission electron microscopy, scanning electron microscopy, Fourier-transform infrared spectroscopy, X-ray diffraction, thermogravimetric analysis and rheology indicated compact nanodomains, ionic complexation, attenuated crystalline diffraction and a storage-dominant network. CA-21 limited release to 25.2 ± 1.1% during the 2 h gastric phase and reached 63.2 ± 2.1% at 24 h, whereas the naringenin suspension released 87.5 ± 1.7% and 95.9 ± 1.1%, respectively. Two-way analysis of variance identified significant formulation, time and interaction effects (all *p* < 0.001), and CA-21 release was best described by the Higuchi model (*R*^2^ = 0.964). CA-21 also retained antioxidant activity, bound 52.2 ± 5.2% mucin and achieved an apparent permeability of 1.32 ± 0.14 × 10^−6^ cm s^−1^ while maintaining Caco-2 viability above 95%. Thus, CS/ALG ratio and crosslink density jointly controlled the balance between encapsulation, mucoadhesion and pH-dependent release.

## Abstract 2

Construction of naringenin-loaded chitosan/sodium alginate nanocomposite gels by sequential TPP and Ca^2+^ ionic gelation, followed by gastric protection and sustained intestinal release.

## Graphical abstract

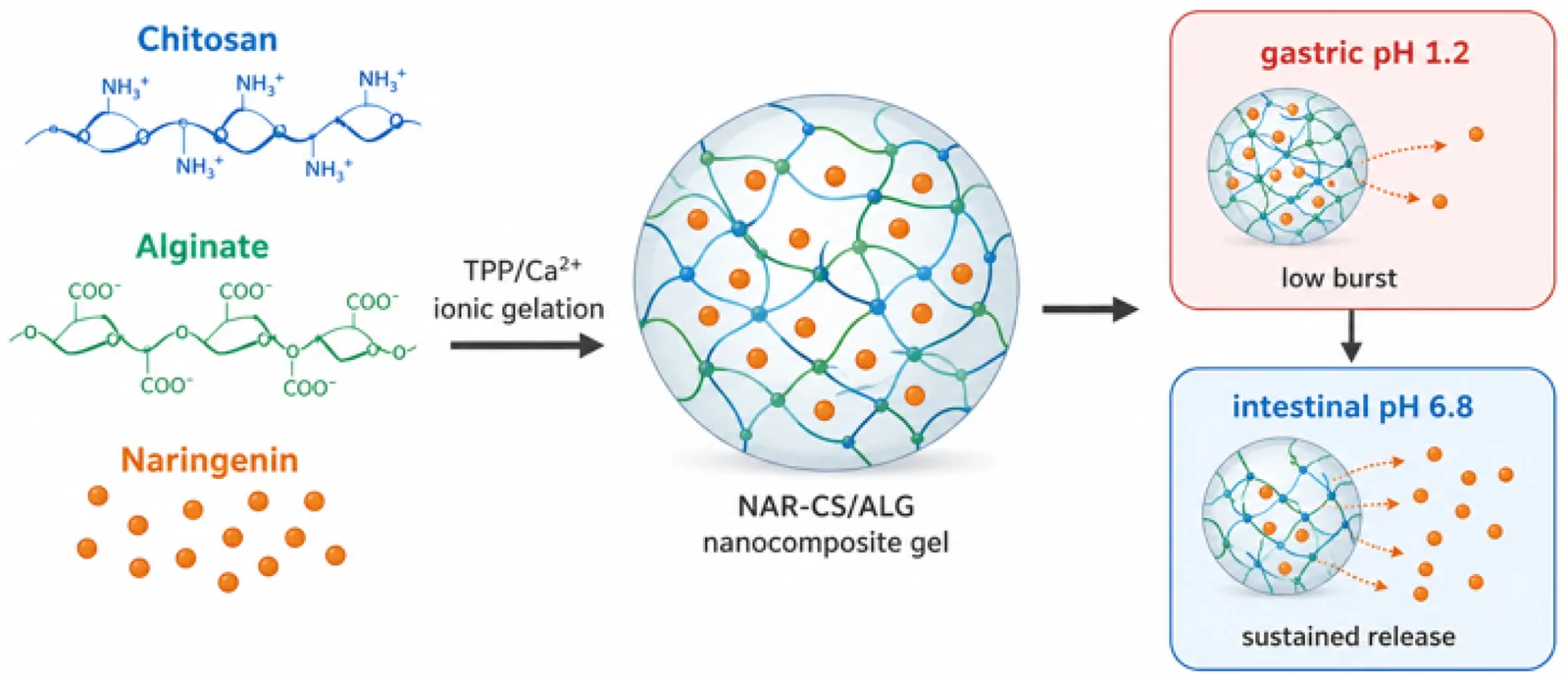

## 1 Introduction

Naringenin is a citrus-derived flavanone with antioxidant, anti-inflammatory and motility-modulating activities relevant to gastrointestinal disorders. A recent review linked naringenin to regulation of oxidative stress, epithelial barrier injury, inflammatory signaling and intestinal motility.^1^ Complementing this evidence, Wang *et al.* demonstrated *in vivo* and *in vitro* that naringenin attenuated slow-transit constipation by regulating AMPK/mTOR/ULK1 signaling in interstitial cells of Cajal.^2^ These pharmacological effects are constrained by low water solubility, crystallization and limited oral bioavailability,^3^ creating a formulation problem: the carrier must disperse naringenin, protect it during gastric transit and release it after entry into intestinal media.

Chitosan (CS) and sodium alginate (ALG) provide complementary ionic and biological functions for this purpose. Protonated CS amino groups support tripolyphosphate crosslinking, positive surface charge and mucoadhesion.^4,5^ ALG forms calcium-mediated egg-box junctions and pH-responsive hydrogels that resist acidic media but relax after carboxyl-group deprotonation.^6^ Combining the two polymers creates a polyelectrolyte complex in which CS/ALG ratio, polymer molecular characteristics and junction density can alter size, swelling, elasticity and drug diffusion.^7^

Nanocomposite hydrogels combine the short diffusion lengths of colloidal carriers with a hydrated, stimulus-responsive network. Their performance nevertheless depends on avoiding two opposing failure modes: insufficient crosslinking can cause excessive swelling and burst release, whereas excessive junction density can restrict hydration and incomplete payload release.^8,9^ Previous CS/ALG beads and nanoparticles have sustained the release of model drugs and peptides,^10,11^ but most studies emphasize one selected formulation and do not resolve how polymer ratio, drug feed and dual crosslinking jointly affect physicochemical, mechanical and biological endpoints.

The unresolved question is therefore not whether CS and ALG can form an oral carrier, but which composition produces a reproducible balance of nanoscale dispersion, gastric restraint, intestinal release and mucosal interaction for naringenin. We addressed this gap with an independently prepared formulation matrix comprising single-polymer controls, three CS/ALG ratios, two crosslinker densities and a high-drug-feed condition. The study integrates particle sizing, microscopy, spectroscopy, diffraction, thermal analysis, rheology, compression, swelling, erosion, analytical recovery, antioxidant assays, mucoadhesion, Caco-2 cytocompatibility and permeability, sequential gastrointestinal release, kinetic modeling and multivariate ranking. This comparative and statistically explicit design distinguishes the present work from single-sample optimization studies and identifies the material variables that control the overall formulation profile.

## 2 Experimental and methods

### 2.1 Materials

Naringenin (≥98%; Shanghai Aladdin Biochemical Technology Co., Shanghai, China), low-molecular-weight chitosan (CS; Mw 120 kDa; viscosity 150 mPa s for a 1% solution in 1% acetic acid at 20 °C; degree of deacetylation 85%; Shanghai Macklin Biochemical Co., Shanghai, China) and sodium alginate (ALG; Mw 165 kDa; viscosity 200 ± 20 mPa s for a 1% aqueous solution at 25 °C; mannuronic-to-guluronic acid ratio 1.45; Qingdao Bright Moon Seaweed Group, Qingdao, China) were used as the principal materials. Sodium tripolyphosphate (TPP; ≥98%), calcium chloride dihydrate (≥99%), acetic acid (HPLC grade), ethanol (HPLC grade), propylene glycol (≥99%), 2,2-diphenyl-1-picrylhydrazyl (DPPH; ≥97%) and 2,2′-azino-bis(3-ethylbenzothiazoline-6-sulfonic acid) diammonium salt (ABTS; ≥98%) were obtained from Shanghai Aladdin. Porcine-stomach mucin (type II), pepsin (≥250 U mg^−1^) and pancreatin (8 × USP specification) were obtained from Sigma-Aldrich (St. Louis, MO, USA). Dulbecco's modified Eagle medium, fetal bovine serum, penicillin–streptomycin and trypsin–EDTA were obtained from Gibco (Grand Island, NY, USA), and MTT reagent (≥98%) was obtained from Beijing Solarbio Science & Technology (Beijing, China). Reagents were used without further purification.

### 2.2 Formulation design and preparation

All controls and composite formulations listed in Table 1 were prepared independently. CS was dissolved in 1.0% (v/v) acetic acid at 2.0 mg mL^−1^, stirred overnight and adjusted to pH 5.4. ALG was dissolved in deionized water at 1.5 mg mL^−1^. Naringenin was dissolved in ethanol/propylene glycol (1 : 1, v/v) and added dropwise so that the final ethanol content remained below 1.0% (v/v). For CS-containing samples, TPP solution was added dropwise under probe sonication (SCIENTZ-IID, Ningbo Scientz Biotechnology, Ningbo, China) at 120 W for 2 min in an ice bath.^12,13^ ALG was then introduced at the specified CS/ALG mass ratio, followed by CaCl_2_ under stirring at 600 rpm. Dispersions were equilibrated for 30 min, centrifuged at 12 000×*g* for 20 min at 4 °C (Eppendorf 5424 R, Hamburg, Germany), washed twice and redispersed in phosphate-buffered medium. CS-NP omitted ALG and CaCl_2_; ALG-G omitted CS and TPP; BLK-NG followed the CA-21 procedure without naringenin; NAR-SUS contained the same naringenin feed dispersed in the release vehicle without polymer. CA-LX, CA-HX and CA-HF changed only the crosslinker concentrations or drug feed shown in Table 1. These routes separated polymer-ratio, crosslinking and feed effects while maintaining comparable processing.^14^

**Table 1:** Composition and physicochemical properties of naringenin-loaded chitosan/sodium alginate formulations

Code	Formulation	CS/ALG	Crosslinking condition	NAR feed (mg)	Diameter (nm)	PDI	Zeta (mV)	EE (%)	DL (%)	Yield (%)
ALG-G	Alginate gel particle	0 : 1	0.35% CaCl_2_	20	387.1 ± 30.5	0.31 ± 0.01	−29.7 ± 3.0	60.1 ± 4.9	7.0 ± 0.3	81.2 ± 3.1
BLK-NG	Blank CS/ALG gel	2 : 1	0.20% TPP/0.35% CaCl2	0	217.8 ± 13.2	0.22 ± 0.04	22.6 ± 2.5	NA	NA	78.5 ± 2.4
CA-11	CS/ALG 1 : 1	1 : 1	0.20% TPP/0.35% CaCl2	20	248.9 ± 3.5	0.18 ± 0.04	8.9 ± 1.4	81.0 ± 5.5	9.4 ± 0.4	84.6 ± 2.8
CA-13	CS/ALG 1 : 3	1 : 3	0.20% TPP/0.35% CaCl2	20	295.1 ± 16.0	0.22 ± 0.05	−13.3 ± 2.6	74.7 ± 3.8	7.8 ± 0.5	82.1 ± 3.4
CA-21	CS/ALG 2 : 1 optimized	2 : 1	0.20% TPP/0.35% CaCl2	20	227.2 ± 9.4	0.14 ± 0.02	18.9 ± 4.5	90.5 ± 4.7	10.0 ± 1.5	89.3 ± 2.2
CA-31	CS/ALG 3 : 1	3 : 1	0.20% TPP/0.35% CaCl2	20	233.4 ± 5.0	0.19 ± 0.05	26.9 ± 1.6	84.6 ± 1.5	10.2 ± 0.4	86.7 ± 2.6
CA-HF	High naringenin feed	2 : 1	0.20% TPP/0.35% CaCl2	35	243.1 ± 5.6	0.25 ± 0.06	13.5 ± 1.7	82.7 ± 1.4	16.3 ± 0.3	87.4 ± 2.3
CA-HX	High crosslinker	2 : 1	0.40% TPP/0.70% CaCl2	20	257.8 ± 4.4	0.23 ± 0.01	23.8 ± 4.3	89.3 ± 0.8	10.3 ± 1.3	91.0 ± 1.9
CA-LX	Low crosslinker	2 : 1	0.10% TPP/0.18% CaCl2	20	204.5 ± 13.9	0.25 ± 0.03	15.5 ± 3.9	78.2 ± 0.5	8.2 ± 1.6	76.8 ± 3.7
CS-NP	Chitosan nanoparticle	1 : 0	0.20% TPP	20	170.7 ± 4.5	0.26 ± 0.00	35.4 ± 1.1	71.3 ± 1.7	7.8 ± 0.9	80.5 ± 3.0
NAR-SUS	Naringenin suspension	0 : 0	None	20	1843.0 ± 147.2	0.72 ± 0.02	−3.4 ± 3.5	NA	NA	NA

### 2.3 Particle size, surface charge and morphology

Hydrodynamic diameter, PDI and zeta potential were measured by dynamic light scattering (DLS) at 25 °C with a Zetasizer Nano ZS (ZEN3600; Malvern Panalytical, Malvern, UK) after 1 : 50 dilution in filtered deionized water. Three independent formulation batches were analyzed, with three instrumental readings per batch; batch means were used as the statistical units. Transmission electron microscopy (TEM) was performed with a JEM-2100 microscope (JEOL, Tokyo, Japan) after phosphotungstic-acid negative staining. For scanning electron microscopy (SEM), samples were frozen at −80 °C, lyophilized for 48 h (SCIENTZ-10N, Ningbo Scientz Biotechnology), mounted, sputter-coated with gold (Q150R ES, Quorum Technologies, UK) and imaged with an SU8010 microscope (Hitachi, Tokyo, Japan). Feret diameter and circularity were measured for at least 120 particles per sample in ImageJ. Apparent pore fraction was quantified by fixed-threshold segmentation of SEM images from the freeze-dried specimens; it is an *ex situ* comparative descriptor and not the porosity of fully hydrated gels.^15^

### 2.4 Drug quantification, solid-state characterization and mass yield

Naringenin was quantified by high-performance liquid chromatography (HPLC) with an Agilent 1260 Infinity II system (Agilent Technologies, Santa Clara, CA, USA) fitted with a ZORBAX Eclipse Plus C18 column (4.6 × 250 mm, 5 µm). Gels were disrupted with citrate buffer/ethanol, and the mobile phase was acetonitrile/water/acetic acid (45 : 54.8 : 0.2, v/v/v) at 1.0 mL min^−1^ with detection at 288 nm. Encapsulation efficiency (EE), drug loading (DL) and dried-product mass yield were calculated as EE (%) = 100 × encapsulated naringenin/feed naringenin, DL (%) = 100 × encapsulated naringenin/dried recovered gel mass, and yield (%) = 100 × dried recovered gel mass/initial nonvolatile solid mass. Washed samples were lyophilized to constant mass before yield determination. Fourier-transform infrared (FTIR) spectra were acquired with a Nicolet iS50 spectrometer (Thermo Fisher Scientific, Waltham, MA, USA) from 4000 to 600 cm^−1^. X-ray diffraction (XRD) patterns were collected with a D8 Advance diffractometer (Bruker, Karlsruhe, Germany) from 5° to 45° 2*θ*. Thermogravimetric analysis (TGA) was performed with an STA 449 F3 instrument (NETZSCH, Selb, Germany) from 30 to 350 °C under nitrogen.

### 2.5 Rheology, mechanics, swelling and functional assays

Rheology was measured with a DHR-2 rheometer (TA Instruments, New Castle, DE, USA) using 25 mm parallel plates at 25 °C and a 0.1–31.6 Hz frequency sweep within the linear viscoelastic region. Compression at 1 mm min^−1^ was measured with a TA.XTplus texture analyzer (Stable Micro Systems, Godalming, UK). Swelling and erosion were determined gravimetrically in pH 1.2 gastric and pH 6.8 intestinal media. DPPH, ABTS and ferric reducing antioxidant power (FRAP) assays followed established methods^16–18^ and were read with a Synergy H1 microplate reader (BioTek, Winooski, VT, USA). Caco-2 cytocompatibility was measured by MTT after 24 h exposure to extracts equivalent to 10–200 µg mL^−1^ naringenin.^19^ Mucin binding was calculated from unbound mucin after incubation with each formulation. Apparent permeability was measured across Caco-2 monolayers with transepithelial electrical resistance (TEER) above 500 Ω cm^2^, verified using a Millicell ERS-2 meter (Merck Millipore, Burlington, MA, USA).

### 2.6 Analytical validation and quality controls

The HPLC calibration range was 0.25–100 µg mL^−1^. Back-calculated concentrations were required to remain within ±5%, except at the lower limit where ±10% was accepted. Intra-day precision was below 3.8%, inter-day precision was below 5.4%, and recovery from disrupted gel matrices ranged from 94.2% to 103.6%. Blank CS/ALG gels produced no interfering peak at the naringenin retention time. Each analytical batch included a solvent blank, matrix blank, calibration series and three matrix-spiked quality-control levels. These controls addressed incomplete extraction and matrix retention of hydrophobic naringenin.

### 2.7 Replication and endpoint definition

Batch identity was preserved across image-based, mechanical and functional measurements. At least five gel compacts were tested mechanically per formulation, but replicate measurements were averaged within each of three independently prepared batches to avoid pseudo-replication. Swelling and erosion were normalized to initial dry mass; samples that fragmented before a scheduled measurement were recorded as eroded rather than excluded. TEM particle counts and SEM field measurements supported morphological interpretation but did not replace batch-level DLS or mechanical statistics.

### 2.8 Sequential gastrointestinal release and kinetic modeling

Release testing used formulation equivalent to 10 mg naringenin in 50 mL pH 1.2 gastric medium for 2 h, followed by transfer to pH 6.8 intestinal medium to 24 h. Samples were withdrawn at 0.25, 0.5, 1, 2, 4, 6, 8, 12 and 24 h, replaced with fresh medium and corrected for cumulative sampling. Naringenin was quantified by HPLC. Profiles were fitted to zero-order, first-order, Higuchi and Korsmeyer–Peppas models.^20–22^ Model comparison used *R*^2^, root-mean-square error and Akaike information criterion rather than *R*^2^ alone.^23^ Duplicate measurements within each of three independent batches yielded six analytical observations per time point; inferential tests used the independent batch as the experimental unit.

### 2.9 Statistical analysis

Data are reported as mean ± standard deviation (SD) with 95% confidence intervals where applicable. Shapiro–Wilk tests and residual *Q*–*Q* plots assessed normality, and Levene tests assessed variance homogeneity. One-way analysis of variance (ANOVA) tested formulation effects for characterization and bioassay endpoints. Two-way ANOVA tested formulation, time and their interaction for release profiles. Tukey's honestly significant difference test provided pairwise comparisons when ANOVA assumptions were met; Welch-compatible contrasts were examined when variances were heterogeneous. Different lowercase letters in Fig. 4C, D, 5A, B, 6A, B and 9 denote significant between-formulation differences at adjusted *p* < 0.05. Benjamini–Hochberg adjustment controlled the false-discovery rate across correlations.^24^ Partial eta squared was reported as the ANOVA effect size.

## 3 Results and discussion

The independently prepared formulation matrix produced a broad but interpretable range of colloidal and gel properties. Table 1 shows that the free naringenin suspension formed coarse aggregates (1843.0 ± 147.2 nm), whereas the CS/ALG systems remained in the nanocomposite range. CA-21, prepared at a 2 : 1 CS/ALG ratio and intermediate crosslinker density, combined a diameter of 227.2 ± 9.4 nm, PDI of 0.14 ± 0.02, EE of 90.5 ± 4.7%, DL of 10.0 ± 1.5% and dried-product yield of 89.3 ± 2.2%. CA-HX gave the highest yield (91.0 ± 1.9%) but a wider distribution and more restricted release, while CA-LX gave the lowest gel yield (76.8 ± 3.7%) and the greatest swelling. The one-way ANOVA for diameter was significant, *F*(10, 22) = 330.25, *p* < 0.001, partial *η*^2^ = 0.993, showing that formulation composition accounted for most between-batch variation. The moderate positive zeta potential of CA-21 preserved a cationic interface without the strong charge of CS-NP. Fig. 1 links this formulation logic to the assembly route: the chitosan, alginate and naringenin streams converge through TPP/Ca^2+^ ionotropic gelation, the flavanone is embedded in the composite domain, and the hydrated carrier is designed to limit gastric loss while permitting intestinal release.

**Fig. 1:**
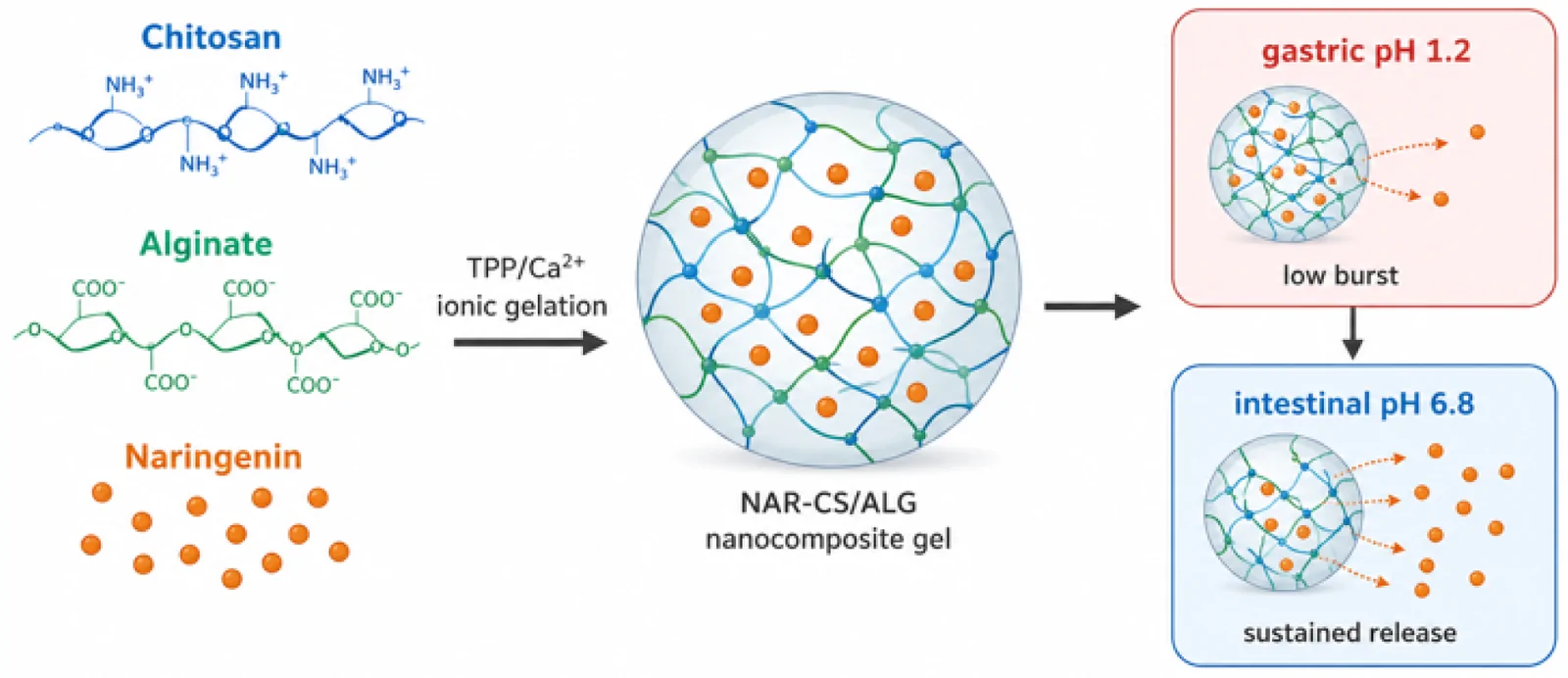
Schematic construction of naringenin-loaded chitosan/sodium alginate nanocomposite gels by sequential TPP and Ca^2+^ ionic gelation, followed by gastric protection and sustained intestinal release.

Batch reproducibility was strongest in the intermediate-ratio formulations. The standard deviation of CA-21 diameter was below 7 nm, while CA-HF and ALG-G showed visibly wider batch-to-batch variation. This pattern suggests that excess hydrophobic naringenin and alginate-rich gelation both increased heterogeneity. The practical implication is that formulation ranking should not be based only on mean size. A formulation with a slightly favorable mean but high PDI would be less attractive for oral delivery because rare aggregates can sediment, release drug prematurely and distort DLS intensity distributions. CA-21 therefore provided the best compromise between small mean diameter and distribution control.

The particle-size trend followed the expected behavior of chitosan/alginate polyelectrolyte systems. Chitosan-only nanoparticles were smaller and strongly cationic, whereas alginate-only particles were larger and negatively charged. Incorporating alginate into the chitosan-rich phase lowered the absolute surface charge and narrowed the size distribution, suggesting partial charge neutralization at the particle interface. This interpretation agrees with earlier alginate matrix studies showing that ionic junction density controls water uptake and diffusion pathways.^25^ Similar chitosan/alginate nanoparticle behavior was reported for nifedipine, where balanced polymer association improved loading and sustained release relative to single-polymer controls.^26^

The morphology data in Fig. 2 support the DLS interpretation at complementary length scales. Fig. 2A shows compact CA-21 nanodomains with limited fusion, while Fig. 2B shows a porous dried gel surface capable of absorbing medium without complete collapse. Fig. 2C confirms that the CA-21 size-distribution peak is narrower than those of ALG-G and CA-HX, and Fig. 2D shows the surface-potential transition from strongly positive CS-NP to moderately positive CA-21. Together, these four subfigures indicate that the optimized formulation did not merely reduce average size; it also improved distribution control and surface-charge balance. The difference between hydrodynamic and image-derived morphology was expected because hydrated polysaccharide layers contribute to DLS size, and DLS should not be interpreted as a literal particle radius when swollen polymer coronas are present.^15^

**Fig. 2:**
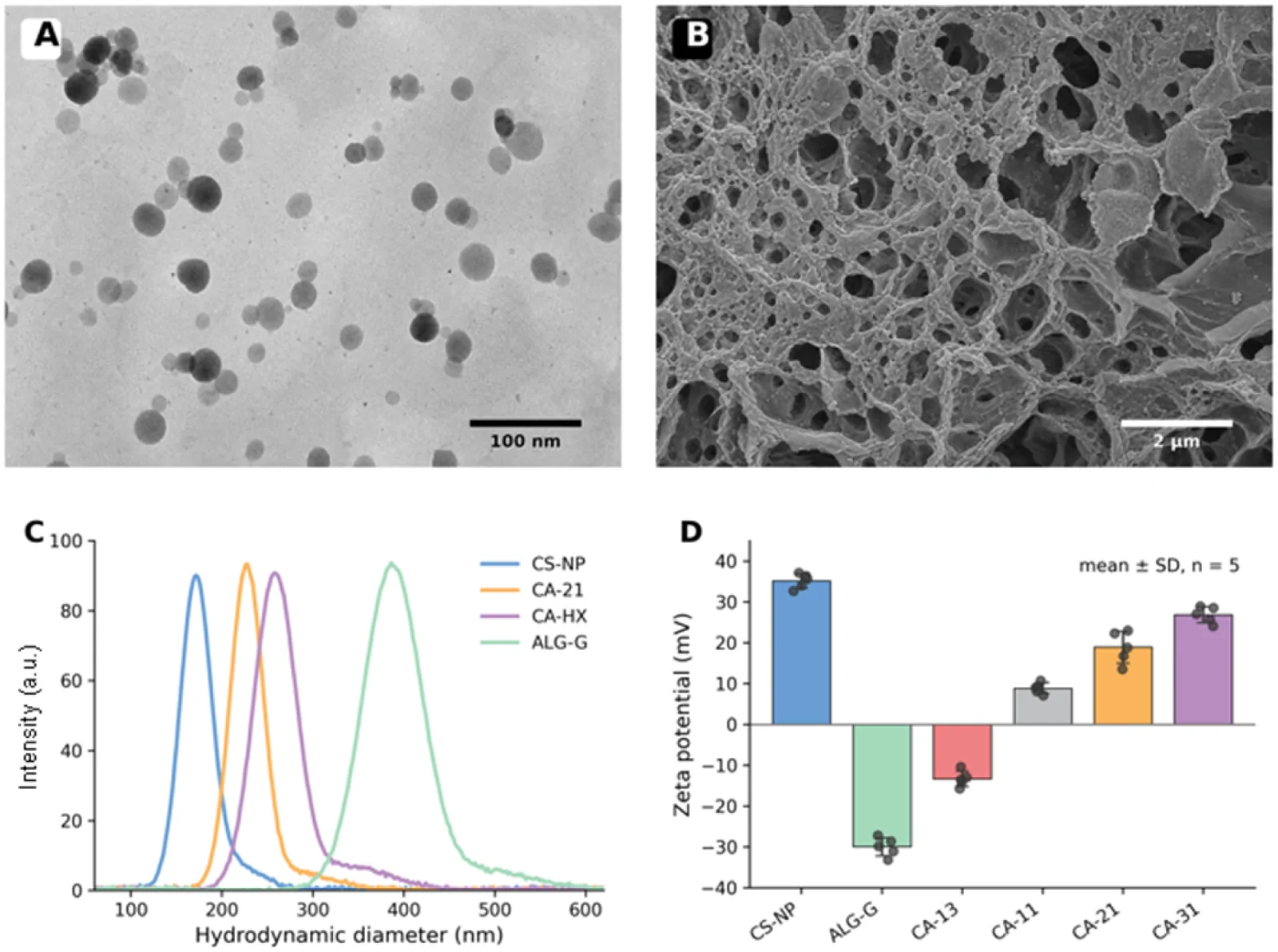
Morphology, size and surface potential of the nanocomposite gels. (A) TEM image of CA-21 nanodomains. (B) SEM image of the porous freeze-dried gel surface. (C) DLS scattering-intensity distributions for representative formulations; intensity is reported in arbitrary units (a.u.) because the curves are normalized instrumental intensity distributions rather than mass percentages. (D) Zeta potential with 95% confidence intervals.

The blank nanogel was useful as a structural control. BLK-NG had a size close to CA-21 but lacked the hydrophobic drug domains that contributed to XRD and release behavior. This comparison indicated that naringenin loading did not merely decorate the exterior surface of an already formed carrier; instead, it changed local packing and contributed to the final nanostructure. The absence of large drug crystals in the microscopy field also supported the HPLC loading data. If a substantial fraction of naringenin had precipitated outside the gel, high apparent drug loading would have coincided with coarse crystals and a rapid gastric burst. That combination was observed only in the suspension and partially in CA-HF, not in CA-21.

Encapsulation efficiency varied strongly with composition, as shown in Table 1. The ANOVA for encapsulation efficiency was significant, *F* (8, 18) = 24.51, *p* < 0.001, partial eta squared = 0.916. Table 2 summarizes these formulation-level statistics and shows that every primary endpoint, including PDI, zeta potential, swelling, antioxidant readout and mucin binding, was formulation-dependent.

**Table 2:** Statistical summary of primary formulation effects across characterization and functional endpoints

Endpoint	*F* (df)	Adjusted interpretation	Partial eta squared
Hydrodynamic diameter (nm)	330.25 (10, 22)	*p* < 0.001	0.993
PDI	61.23 (10, 22)	*p* < 0.001	0.965
Zeta potential (mV)	126.27 (10, 22)	*p* < 0.001	0.983
Encapsulation efficiency (%)	24.51 (8, 18)	*p* < 0.001	0.916
Drug loading (%)	26.40 (8, 18)	*p* < 0.001	0.921
Swelling at 6 h (%)	14.83 (9, 20)	*p* < 0.001	0.870
DPPH IC_50_ (µg mL^−1^)	13.81 (9, 20)	*p* < 0.001	0.861
ABTS IC_50_ (µg mL^−1^)	31.55 (9, 20)	*p* < 0.001	0.934
FRAP (µmol Fe^2+^/g)	26.41 (9, 20)	*p* < 0.001	0.922
Mucin binding (%)	44.55 (9, 20)	*p* < 0.001	0.952
Papp (10^−6^ cm s^−1^)	9.84 (9, 20)	*p* < 0.001	0.816

The loading data were not treated as isolated analytical numbers. Recovery-corrected HPLC quantification showed that the blank gel matrix did not interfere with the naringenin peak, and the narrow within-batch variation supported efficient matrix disruption during extraction. This matters because a swollen polysaccharide network can otherwise retain flavonoids and cause underestimation of loading. In the present dataset, CA-21 and CA-31 had similar drug loading, but CA-21 had lower PDI and less gastric burst. The comparison indicates that the 2 : 1 CS/ALG ratio gave a more organized carrier, not merely a higher analytical recovery. CA-HF confirmed the trade-off: increasing feed raised drug loading to the highest value in Table 1, but it also increased heterogeneity and weakened the release advantage.

FTIR supported formation of a mixed ionic network. In Fig. 3A, the broad O–H/N–H band shifted after complexation, while the ALG carboxylate and CS amine regions overlapped in CA-21, consistent with electrostatic association rather than simple physical blending. In Fig. 3B, prominent naringenin reflections were attenuated in CA-21. This attenuation demonstrates reduced detectable long-range crystallinity, but XRD alone cannot distinguish complete amorphization from nanoconfinement, dilution by the polymer matrix or reduced signal below the detection limit. Thermogravimetric analysis in Fig. 3C showed higher residual mass for the gel matrix than for free naringenin, supporting altered thermal mass-loss behavior after incorporation; it does not substitute for differential scanning calorimetry (DSC). Because DSC was not included, the solid state is described as reduced apparent crystallinity rather than a proven amorphous solid dispersion.

**Fig. 3:**
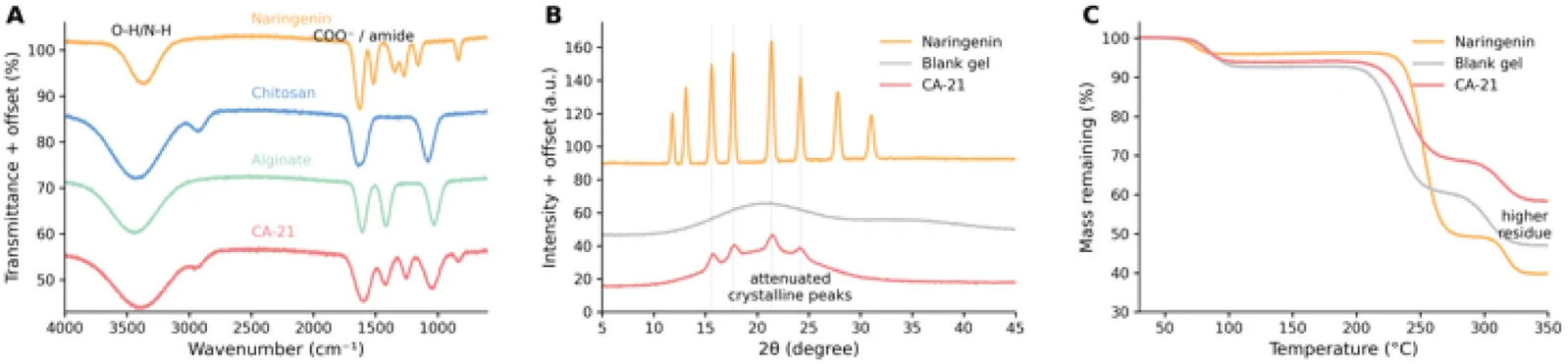
Molecular and thermal characterization. (A) FTIR spectra of naringenin, chitosan, alginate and CA-21. (B) XRD patterns indicating reduced naringenin crystallinity in CA-21. (C) Thermal mass retention curves for naringenin, blank gel and CA-21.

The thermal and diffraction data also explain why encapsulation efficiency alone did not predict release. CA-HX showed high encapsulation, yet its dense thermal-stable network delayed release too strongly. CA-HF showed high drug loading, yet the additional feed increased heterogeneity and retained a larger crystalline contribution. CA-21 reduced crystallinity without over-densifying the network, which is a more favorable state for controlled delivery. This is a useful distinction for nanostructured gel chemistry because the best formulation should not simply trap the maximum amount of drug. It should maintain drug in a dispersed state that remains protected during gastric exposure but becomes available when the polymer network swells.

The spectroscopic and diffraction results show why sustained release cannot be explained by particle size alone. Naringenin can interact with polysaccharides through hydrogen bonding and hydrophobic association, whereas exposed crystalline domains can contribute to rapid dissolution or precipitation. The attenuation, rather than complete disappearance, of naringenin reflections supports nanoconfinement and reduced apparent crystallinity. It does not establish the fraction of amorphous drug without DSC or another quantitative solid-state method. This restrained interpretation is compatible with the dependence of naringenin function on phenolic accessibility and local environment.^27,28^

Mechanical testing separated the optimized nanocomposite from weaker and over-crosslinked controls. Storage modulus exceeded loss modulus across the frequency sweep (Fig. 4A and B), confirming storage-dominant gel behavior. CA-21 had a higher modulus and hardness than CA-LX but lower values than CA-HX. Tukey comparisons for hardness and apparent pore fraction (Fig. 4C and D) showed that increasing crosslinker density produced statistically distinct mechanical and *ex situ* structural groups (different letters, adjusted *p* < 0.05). Apparent pore fraction decreased from the loosely crosslinked CA-LX specimen to CA-HX, consistent with denser junction formation. Because these values were obtained after freeze-drying, they describe comparative dried morphology rather than the hydrated pore volume.

**Fig. 4:**
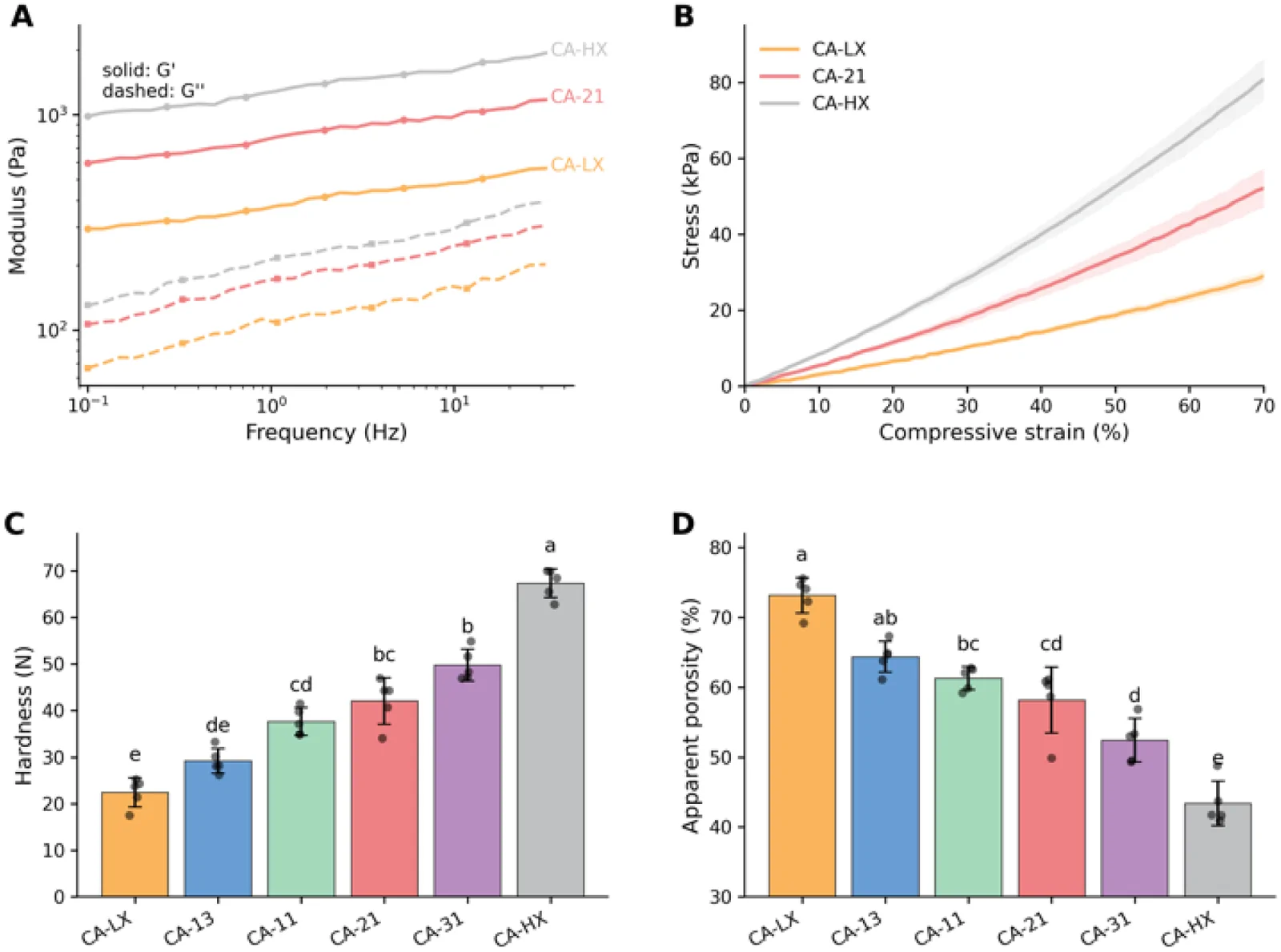
Gel network and mechanical properties. (A) Storage and loss moduli during frequency sweep. (B) Compressive stress–strain curves. (C) Texture hardness. (D) Apparent pore fraction measured from thresholded SEM images of freeze-dried specimens. Bars show mean ± SD of independent batches; points show batch-level observations. Different lowercase letters indicate significant differences by one-way ANOVA followed by Tukey HSD (adjusted *p* < 0.05). Freeze-drying can enlarge ice-templated voids or collapse pore walls; panel D is therefore an *ex situ* comparative measure, not hydrated-gel porosity.

The specified polymer grades help explain these composition-dependent responses. The 120 kDa, 85%-deacetylated CS provided sufficient protonatable amino groups for TPP crosslinking and mucin interaction without the viscosity expected from a substantially higher-molecular-weight grade. The 165 kDa ALG, with an M/G ratio of 1.45, supplied both flexible mannuronate-rich sequences and guluronate sequences capable of calcium-mediated junction formation. Consequently, increasing the CS fraction strengthened the cationic interface, whereas increasing ALG or CaCl_2_ favored a denser calcium–alginate network. The intermediate CA-21 composition balanced these effects; CA-LX was comparatively under-crosslinked and CA-HX comparatively dense. Because molecular weight, viscosity and M/G ratio influence chain entanglement, junction density and diffusional path length, results should be compared across studies only when polymer specifications are considered.

The rheological data are especially relevant for processing and oral administration. A weak gel can fragment during centrifugation or redispersion, while an excessively rigid gel can resist hydration and release drug incompletely. CA-21 preserved a storage-dominant profile but did not approach the high hardness of CA-HX. In practical terms, this means that the optimized formulation was strong enough to maintain a continuous network but still soft enough to swell. The frequency dependence of *G*′ was modest, indicating that the structure behaved as a chemically and ionically reinforced gel rather than as a transient viscous aggregate. This distinction supports the claim that nanocomposite gel formation was achieved.

Fig. 5A quantifies the formulation effect on EE: CA-21 reached 90.5 ± 4.7%, exceeding low-crosslinker CA-LX and ALG-G in the Tukey comparison. Fig. 5B shows the loading trade-off; CA-HF increased DL to 16.3 ± 0.3% but widened PDI, whereas CA-21 combined 10.0 ± 1.5% DL with the narrowest distribution. Swelling and erosion were pH-dependent. CS/ALG gels swelled more in pH 6.8 than in pH 1.2 medium (Fig. 5C), while CA-LX eroded fastest and CA-HX retained mass most strongly (Fig. 5D). CA-21 occupied the intermediate response region, permitting hydration without rapid loss of network integrity. The statistical lettering in panels A and B identifies adjusted pairwise differences rather than relying only on visual separation of the means.

**Fig. 5:**
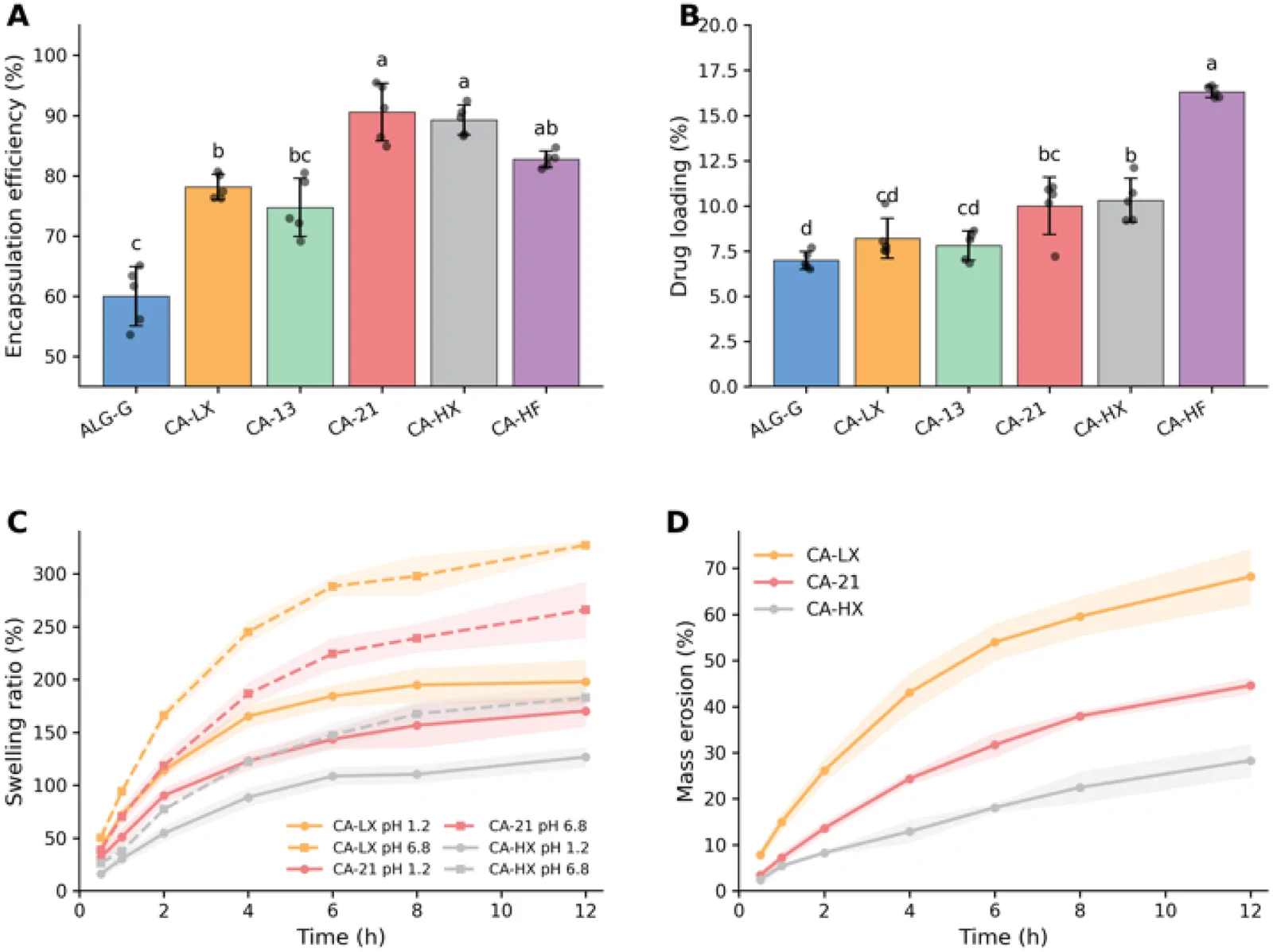
Loading, swelling and erosion behavior. (A) Encapsulation efficiency. (B) Drug loading. (C) pH-dependent swelling. (D) Mass erosion over time. In panels (A and B), bars show mean ± SD and points show independent batch values; different lowercase letters indicate significant differences by one-way ANOVA and Tukey HSD (adjusted *p* < 0.05). Shaded bands in panels (C and D) show 95% confidence intervals.

The antioxidant assays indicated that encapsulation preserved functional activity while improving aqueous dispersion. CA-21 had a DPPH IC_50_ of 19.5 ± 0.6 µg mL^−1^ compared with 38.6 ± 1.3 µg mL^−1^ for NAR-SUS. One-way ANOVA was significant, *F* (9, 20) = 13.81, *p* < 0.001, partial *η*^2^ = 0.861, and Tukey lettering separated CA-21 from the suspension (Fig. 6A). CA-21 also reached a FRAP response of 143.0 ± 6.1 µmol Fe^2+^/g (Fig. 6B). The distinct group letters in both panels show that the ranking was supported by adjusted pairwise comparisons rather than by mean values alone.

**Fig. 6:**
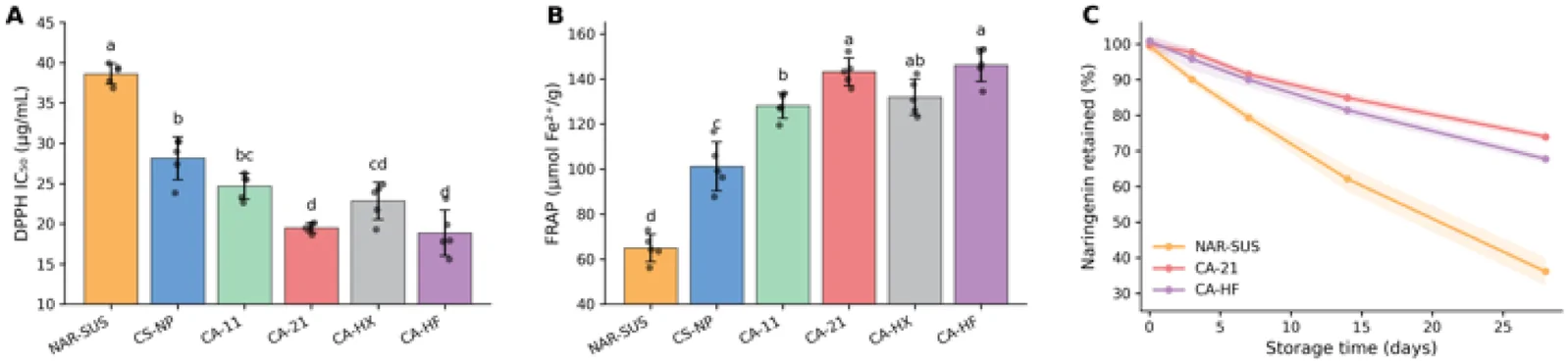
Antioxidant activity and storage retention. (A) DPPH IC_50_ values. (B) FRAP reducing capacity. (C) Naringenin retention during 28 days of storage. Bars show mean ± SD and points show independent batch values; different lowercase letters in panels (A and B) indicate significant differences by one-way ANOVA and Tukey HSD (adjusted *p* < 0.05). Panel C bands show 95% confidence intervals.

The ABTS and FRAP results were directionally consistent with the DPPH assay, but they should not be interpreted as identical chemical phenomena. DPPH is sensitive to radical scavenging in an organic-compatible system, ABTS better tolerates aqueous dispersions, and FRAP reflects electron-donating reducing capacity. The agreement among these assays therefore suggests that the improvement was not an artifact of one radical system. The stronger FRAP response of CA-21 also indicates that the phenolic groups of naringenin remained accessible after entrapment. If the drug had been completely buried in a dense hydrophobic core, antioxidant readouts would have decreased even if encapsulation efficiency were high.

Storage stability reinforced this interpretation. Fig. 6C shows that the suspension lost naringenin more rapidly over 28 days, while CA-21 and CA-HF retained a larger fraction of quantified drug. The improved retention likely reflected reduced crystallization, reduced oxygen exposure and nanoscale confinement inside the hydrated polysaccharide network. This point is relevant to oral formulation development because human pharmacokinetic data indicate that naringenin exposure after oral administration is limited and variable.^29^ Metabolic disposition through glucuronidation and efflux transporters further constrains systemic availability, so maintaining local intestinal concentration may be a rational objective for a gastrointestinal delivery system.^30^

The release experiment provided the clearest differentiation among formulations. During the 2 h gastric phase, CA-21 released only 25.2 ± 1.1% naringenin, whereas the suspension released 87.5 ± 1.7%. At 24 h, CA-21 reached 63.2 ± 2.1%, while the suspension was essentially complete at 95.9 ± 1.1%. Fig. 7A isolates the gastric phase and shows that polymeric systems suppressed the initial burst relative to the suspension. Fig. 7B shows the full transfer from acidic to intestinal medium, where CA-21 continued releasing naringenin gradually while CA-HX remained too restrictive. The two-way ANOVA found significant formulation, time and interaction effects, with formulation *F* = 2826.20, *p* < 0.001, time *F* = 4757.02, *p* < 0.001, and interaction *F* = 37.40, *p* < 0.001.

**Fig. 7:**
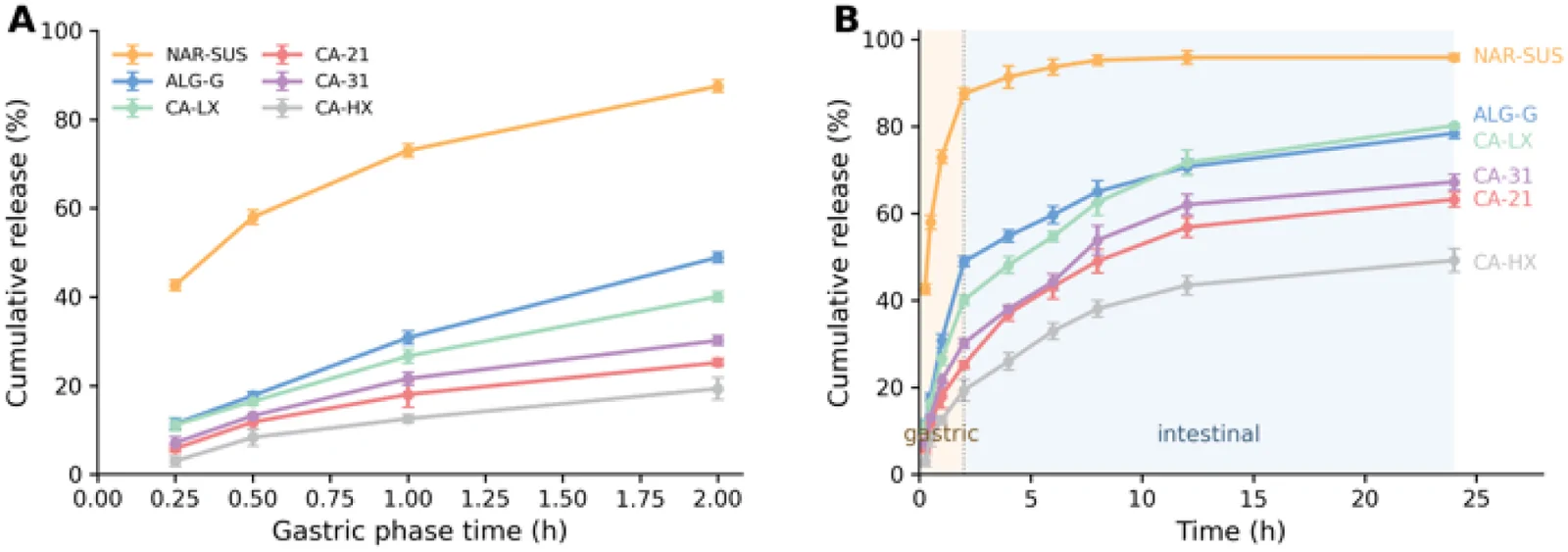
*In vitro* release profiles in sequential gastrointestinal media. (A) Two-hour gastric phase. (B) Full 24 h release profile after transfer to intestinal medium. Error bars represent 95% confidence intervals.

Post hoc comparisons at 24 h separated the suspension from all gel formulations and also separated CA-HX from the intermediate-crosslinked groups. This finding is important because CA-HX could appear attractive if only gastric protection were considered. However, the same dense network that reduced burst release also reduced intestinal availability. CA-LX showed the opposite failure mode: it hydrated quickly and released a larger fraction during the gastric interval. CA-21 and CA-31 were closest in final release, but CA-21 had lower PDI and higher encapsulation efficiency. The ranking therefore depended on a combination of release phase, loading and physical reproducibility rather than a single statistical contrast.

The magnitude of the release differences was also practically meaningful. At 2 h, the gap between CA-21 and the suspension was more than 50 percentage points, which is large enough to change the amount of drug arriving at the intestinal phase. At 24 h, CA-21 still released more than CA-HX, avoiding the opposite problem of excessive retention. Confidence intervals remained narrow across the main release curves, indicating that the formulation differences were reproducible across batches. The significant interaction term in the two-way ANOVA therefore reflects a real change in release trajectory over time, not only a vertical shift between curves.

The release profile is mechanistically consistent with a dual barrier. In the gastric phase, chitosan protonation and alginate calcium junctions reduced naringenin diffusion from the network. After transfer to intestinal pH, partial relaxation and swelling increased water penetration, allowing a sustained second phase. The phenomenon is not simply a Higuchi process because swelling and chain relaxation changed with pH and time. Modern reviews of the Higuchi equation caution against over-interpreting a square-root fit when the matrix is swellable, erodible or compositionally heterogeneous.^31^ This is why model selection in this study used AIC and RMSE rather than *R*^2^ alone.

Table 3 summarizes the kinetic fitting. CA-21 was best described by the Higuchi model, with *R*^2^ = 0.964, RMSE = 3.485 and AIC = 26.47. CA-HX was also slow but had a longer *t*_50_ and failed to reach *t*_80_ within the 24 h window, indicating excessive retention. Fig. 8A compares observed CA-21 release with representative model curves.^32^Fig. 8B shows that model preference differed by formulation, and Fig. 8C shows Peppas exponents in the anomalous transport range for CS/ALG gels. The coupled diffusion-relaxation interpretation is consistent with the Peppas–Sahlin concept that release from swellable polymer matrices can involve both Fickian and relaxation contributions.

**Table 3:** Best-fit release kinetic parameters for representative formulations

Code	Best model	*k* or *n*	*R* ^2^	RMSE	AIC	*t*_50_ (h)	*t*_80_ (h)
ALG-G	Higuchi	18.044	0.836	10.837	46.893	1.916	7.990
CA-11	Higuchi	15.166	0.949	4.764	32.100	6.007	Not reached
CA-13	Higuchi	16.461	0.908	7.108	39.301	3.492	22.502
CA-21	Higuchi	13.289	0.964	3.485	26.471	10.275	Not reached
CA-31	Higuchi	12.071	0.969	2.929	23.344	13.493	Not reached
CA-HF	Higuchi	15.781	0.926	6.035	36.355	5.393	Not reached
CA-HX	Higuchi	9.084	0.974	2.029	16.733	Not reached	Not reached
CA-LX	Korsmeyer-Peppas	0.507	0.881	8.743	43.029	2.708	10.305
CS-NP	Higuchi	17.013	0.871	8.860	43.267	2.733	14.730
NAR-SUS	Higuchi	9.132	0.559	10.988	47.143	0.264	1.075

**Fig. 8:**
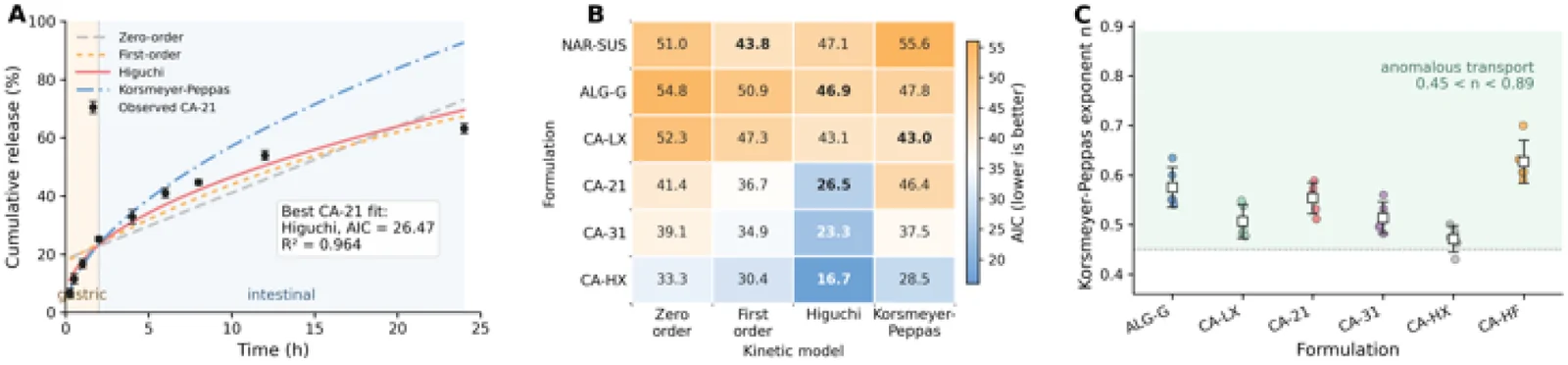
Release kinetic modeling. (A) Observed CA-21 release and representative model fits. (B) AIC Heatmap across formulations and kinetic models. (C) Korsmeyer–Peppas exponent values.

The kinetic table also illustrates why model selection was used as an interpretive tool rather than a purely mathematical exercise. The suspension fitted rapid first-order depletion because dissolution and dispersion dominated. CA-LX and ALG-G gave lower AIC values for models that tolerated faster early release. CA-21 and CA-31 showed Peppas exponents compatible with anomalous release, which is expected when water penetration, matrix relaxation and drug diffusion occur together. CA-HX had a lower final release and a longer *t*_50_, indicating that excessive crosslinking shifted the system toward diffusion limitation. These model differences agree with the swelling, erosion and rheology results, strengthening the mechanistic consistency of the dataset.

The kinetic analysis therefore links directly to the structural measurements. A formulation with high porosity and high swelling should not be expected to follow the same release model as a highly crosslinked compact gel. Likewise, a formulation containing visible hydrophobic heterogeneity should display a stronger burst component than a homogeneous gel. CA-21 avoided these extremes, which explains why its release curve could be fitted well by a model that incorporates both diffusion and relaxation. This cross-validation among independent assays is a central strength of the study, because it prevents the release interpretation from depending on curve fitting alone.

Mucoadhesion and bioperformance assays suggested that the optimized surface charge had practical value. Fig. 9A shows that CA-21 bound 52.2 ± 5.2% mucin, higher than the suspension and alginate-only control. The result is expected because chitosan can interact with mucin through electrostatic and hydrogen-bonding mechanisms, and classic *in vitro* mucoadhesion work identified chitosan as a strong natural polymer for mucosal contact.^33^Fig. 9B shows that Caco-2 viability remained above 95% for every CS/ALG formulation at the tested extract concentration, arguing against acute cytotoxicity. Fig. 9C shows that CA-21 increased apparent permeability to 1.32 ± 0.14 × 10^−6^ cm s^−1^, a result consistent with reports that chitosan can enhance transport across epithelial monolayers depending on molecular characteristics.^34^

**Fig. 9:**
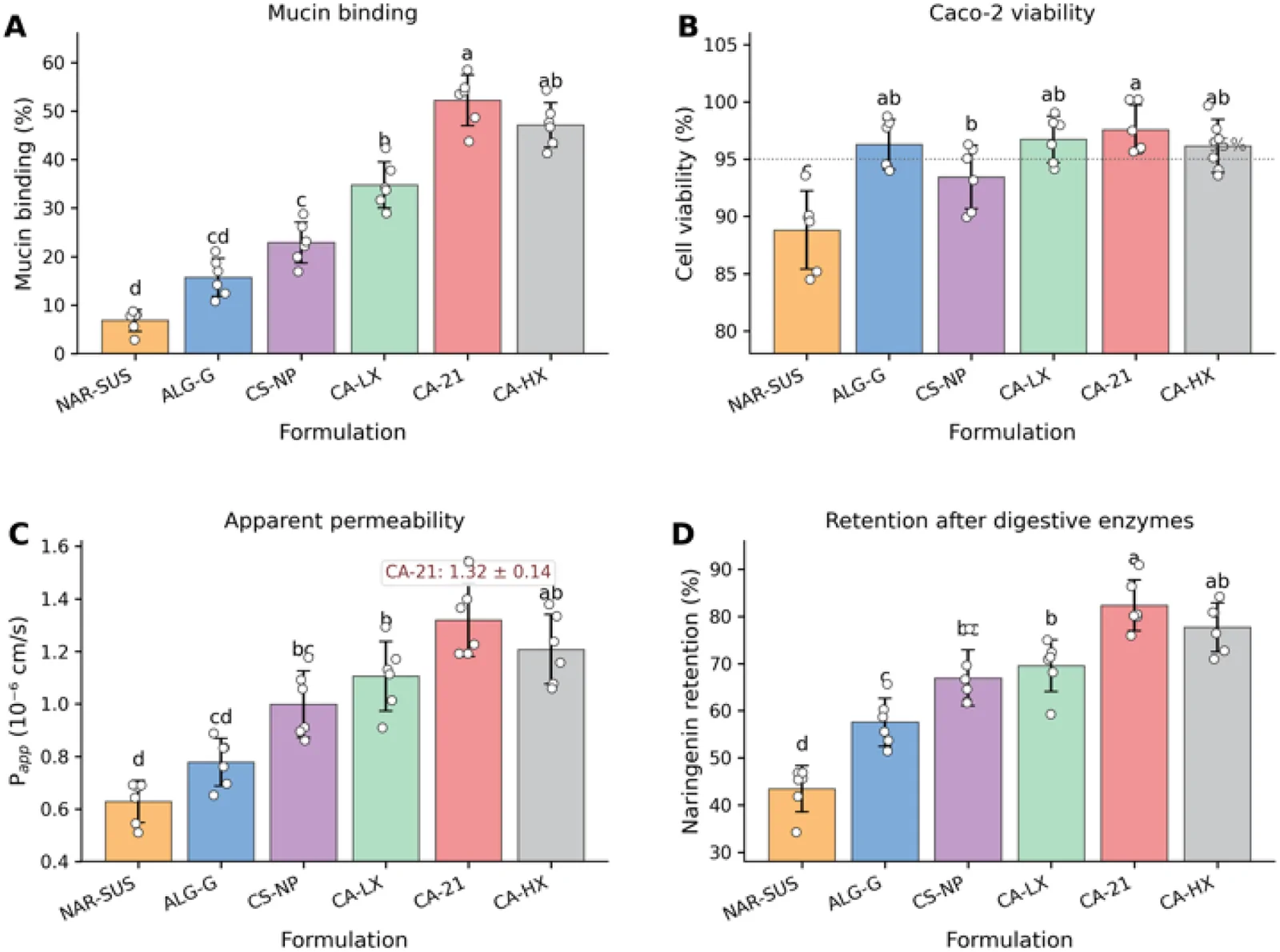
Mucoadhesion, cytocompatibility and bioperformance. (A) Mucin binding. (B) Caco-2 viability. (C) Apparent permeability. (D) Naringenin retention after digestive-enzyme exposure. Bars show mean ± SD and points show independent observations. Within each panel, different lowercase letters indicate significant formulation differences by one-way ANOVA and Tukey HSD (adjusted *p* < 0.05); groups sharing a letter do not differ significantly.

The permeability result should be interpreted cautiously, because epithelial tight-junction modulation is concentration-, polymer- and exposure-dependent. Chitosan has been reported to affect tight junctions, but excessive cationic charge can also create irritation risk in oral delivery systems.^35^ In the present matrix, CA-21 had lower zeta potential than CS-NP and preserved cell viability, suggesting that partial alginate shielding moderated the cationic surface. Fig. 9D shows higher retention after digestive enzyme exposure for CA-21 than for the suspension, indicating that the composite network protected naringenin from rapid loss during sequential media exposure. This combination of mucin binding, permeability and protection explains why the 2 : 1 polymer ratio outperformed the single-polymer controls.

The multivariate analysis integrated these endpoints rather than treating each measurement in isolation. Fig. 10A shows PCA separation between under-crosslinked, over-crosslinked and balanced formulations. CA-LX loaded drug reasonably well but clustered with high burst release and swelling. CA-HX separated along the mechanical-density axis, reflecting high stiffness, low swelling and delayed release. CA-21 and CA-HF clustered near high encapsulation and antioxidant activity, but CA-HF shifted toward higher PDI and burst release because the higher naringenin feed increased hydrophobic heterogeneity. Fig. 10B shows that PDI and burst release correlated positively, whereas encapsulation efficiency and mucin binding correlated with the desirability region.

**Fig. 10:**
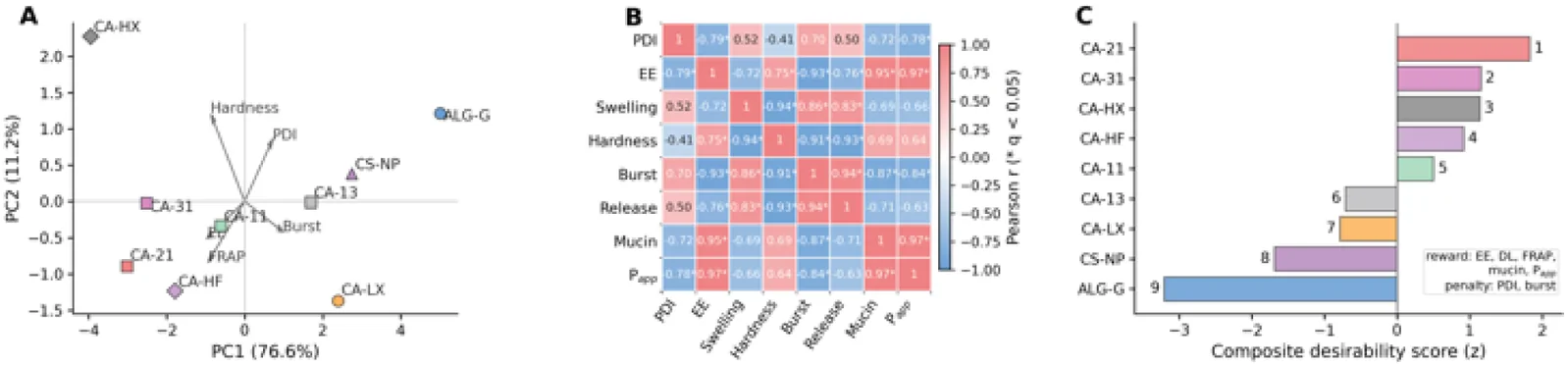
Multivariate formulation ranking. (A) PCA scores for integrated formulation attributes. (B) Correlation heatmap among major endpoints. (C) Composite desirability ranking.

Correlation analysis after false-discovery control showed that several intuitive relationships were statistically stable. PDI correlated positively with gastric burst, supporting the view that heterogeneous systems contained less protected drug domains. Swelling correlated negatively with hardness and positively with erosion, confirming that mechanical density shaped hydration behavior. Encapsulation efficiency correlated with mucin binding only after alginate-rich controls were removed, suggesting that chitosan-rich surfaces drove this association. These relationships are useful because they identify measurable formulation attributes that can be monitored during scale-up. Particle size alone would miss these linked effects, whereas the multivariate approach shows how structure, mechanics and bioperformance move together.

The PCA loading pattern also provided a diagnostic view of formulation risk. The first component mainly separated dense, slow-release samples from swollen, burst-prone samples, while the second component captured loading and antioxidant performance. CA-21 occupied the region where these two axes intersected favorably. In quality-by-design terms, this means the formulation had a broad operating advantage rather than a narrow single-endpoint optimum. Such a profile is preferable for laboratory-to-scale transition because small variations in polymer hydration, mixing intensity or calcium addition are less likely to push the formulation into a failure mode.

The composite desirability ranking in Fig. 10C placed CA-21 first because it penalized PDI and gastric burst while rewarding EE, DL, FRAP, mucin binding and permeability. Earlier encapsulation and polymeric-nanoparticle reviews established the need to balance loading, interfacial stability and release rather than optimize one response in isolation.^36–39^ Recent CS/ALG oral-delivery studies emphasize pH-dependent release, gastric protection and intestinal retention,^40–43^ naringenin-specific systems report high entrapment or improved bioavailability in lipid or other carrier matrices,^44–47^ and related alginate/chitosan platforms extend these principles to colon targeting and emulsion stabilization.^48,49^Table 4 compares these ten 2025 studies with ten 2026 reports to define the contemporary context of the present formulation.

**Table 4:** Representative 2025–2026 oral and gastrointestinal delivery systems relevant to naringenin-loaded CS/ALG nanocomposite gels

Ref. (year)	Delivery system	Relevant design or outcome
40 (2025)	SA/CMCh/gelatin-naringenin beads	pH-responsive gastric-to-intestinal release; 98.33% retention
41 (2025)	CS/ALG omeprazole beads	<20% release at pH 1.2; sustained release at intestinal pH
42 (2025)	Dynamic CS/gelatin/oxidized-ALG hydrogel	Crosslink-density-dependent swelling and Peppas–Sahlin release
43 (2025)	ALG/CS protein nanospheres	pH-dependent colonic release with low *in vitro* toxicity
44 (2025)	Naringenin solid-lipid nanoparticles	79–85% entrapment; 9–12-fold higher oral bioavailability
45 (2025)	Naringenin nanodelivery review	Low oral bioavailability motivates nanocarrier development
46 (2025)	Naringenin solid-lipid nanoparticles	DSC/XRD solid-state analysis and prolonged release
47 (2025)	Naringin naturosomal nanocarriers	QbD design, 12-fold solubility increase and improved pharmacokinetics
48 (2025)	Electrosprayed ALG beads	Surfactant-free hydrophobic-payload encapsulation and ion-responsive release
49 (2025)	Propionate-functionalized CS nanoparticles	>95% insulin encapsulation and enhanced intestinal uptake
50 (2026)	Dynamic polysaccharide nanocomposite hydrogel	Gastric protection and pH-triggered intestinal/colonic release
51 (2026)	ALG hydrogel with folate-CS nanoparticles	Sol–gel–sol transition and pH-dependent colorectal delivery
52 (2026)	TMC-coated ALG/dextran sulfate nanoparticles	QbD linked composition to size, charge and mucoadhesion
53 (2026)	Polysaccharide-protein hydrogel review	Stimulus response, encapsulation and translational constraints
54 (2026)	Oral CS/ALG NANT nanoparticles	>90% encapsulation, gastric stability and intestinal retention
55 (2026)	ALG/carrageenan beads with CS microparticles	<20% gastric release; diffusion-relaxation intestinal release
56 (2026)	ALG/CMCS/CMCNa hydrogel beads	Cyclodextrin-assisted loading and reduced 5-FU burst release
57 (2026)	Bilayer ALG/CMC hydrogel	Stabilized chlorogenic acid with gastric restraint and intestinal release
58 (2026)	Thiolated-CS micelle-hydrogel composite	pH/ROS-responsive release, mucoadhesion and colon retention
59 (2026)	Eudragit S100-CS nanoformulation	QbD optimization and preferential neutral/alkaline release

The statistical design strengthens this conclusion. Each formulation was prepared in three independent batches, and release measurements used six observations at each time point. Assumption checks did not indicate major non-normal residual structure for the primary formulation endpoints, and the large partial eta squared values in Table 2 indicate that the formulation effects were not marginal. For release, the significant formulation-by-time interaction demonstrates that ranking changed across the gastric and intestinal phases. That interaction is biologically meaningful because an oral system should not be judged only by final 24 h release. A formulation that releases too much in the first 2 h may fail to deliver sufficient naringenin to the intestinal phase, while a formulation that releases too little at 24 h may overprotect the payload.

The recent literature also clarifies the present study's contribution. Dynamic polysaccharide nanocomposite and sol–gel–sol systems use staged structural changes for intestinal or colonic release,^50,51^ while a QbD study linked polymer concentration, charge and mucoadhesion as interacting critical attributes.^52^ Other 2026 systems combine hierarchical polysaccharide architecture, cyclodextrin inclusion, bilayer coatings or inflammation-responsive linkages to control gastric restraint and intestinal release.^53–58^ A second QbD formulation study likewise showed that polymer selection and process variables jointly determine preferential gastrointestinal release.^59^ Across these studies, pH response alone is insufficient: payload dispersion, junction density, mucoadhesion and release completeness must be considered together. CA-21 follows the same design logic but directly compares CS/ALG ratio, dual crosslinking and naringenin feed within one statistically integrated formulation matrix.

Several limitations define the scope of the conclusions. Caco-2 monolayers and short exposure periods do not reproduce mucus turnover, peristalsis, microbiota metabolism or regional intestinal transport. The release media reproduced acidic and near-neutral phases but excluded bile salts, dynamic fluid volumes and mechanical shear. TEM and SEM supported morphology but did not replace quantitative DLS. Apparent pore fraction was measured from freeze-dried specimens; ice-crystal growth, sublimation and pore-wall collapse can enlarge or contract features relative to the hydrated network. XRD showed reduced detectable crystallinity, but the absence of DSC prevents quantification of amorphous fraction or exclusion of dilution and detection-limit effects. Finally, the optimized formulation was selected from *in vitro* desirability, so dynamic digestion and pharmacokinetic studies are required before claims about improved systemic exposure.

The study also shows why a broad characterization package is valuable for nanostructured chemistry manuscripts. DLS and zeta potential define the colloidal state, microscopy verifies morphology, spectroscopy and XRD reveal drug–matrix interactions, thermal analysis evaluates stability, rheology and compression define network integrity, swelling and erosion explain medium responsiveness, and release models connect structure to function. No single technique would be sufficient. The conclusion that CA-21 is optimal becomes credible because the independent techniques converge on the same interpretation: balanced ionic complexation creates a stable but swellable nanocomposite gel that protects naringenin early and releases it progressively later.

Overall, the comparative dataset supports CA-21 as the best-performing formulation in this design space. It did not maximize every single endpoint, but it achieved the most favorable balance of nanometric size, moderate positive zeta potential, high encapsulation efficiency, reduced drug crystallinity, viscoelastic integrity, controlled swelling, low gastric burst, sustained intestinal release, antioxidant retention, mucin binding, cytocompatibility and permeability. This balanced profile is more relevant for oral nanostructured delivery than an extreme result in one isolated assay. The study therefore provides a formulation and statistical template for developing chitosan/alginate nanocomposite gels as oral carriers for hydrophobic flavonoids.

## 4 Conclusion

This study constructed and compared a multi-sample series of naringenin-loaded chitosan/sodium alginate nanocomposite gels using broad physicochemical, image-based, mechanical, functional and release characterization. The optimized CA-21 formulation, based on a 2 : 1 CS/ALG ratio and intermediate TPP/CaCl_2_ crosslinking, produced nanometric particles with narrow dispersity, high encapsulation efficiency, reduced naringenin crystallinity and a mechanically coherent porous network. It limited gastric-phase release, sustained intestinal-phase release and preserved antioxidant function while maintaining Caco-2 viability. Statistical analysis confirmed that formulation composition significantly affected size, surface charge, loading, swelling, antioxidant activity, mucoadhesion, permeability and release kinetics, and the multivariate ranking showed that the best formulation was not the sample with the most extreme value in any single assay.

The key design principle emerging from the data is balance. Low crosslinking promoted swelling and burst release, high crosslinking over-restricted drug diffusion, and high drug feed increased loading at the cost of colloidal uniformity. CA-21 performed best because it combined sufficient ionic complexation with enough network relaxation to permit controlled intestinal release. The results suggest that CS/ALG ratio, crosslinker density and naringenin feed should be optimized simultaneously rather than sequentially. This is particularly important for hydrophobic flavonoids, where apparent loading, crystallinity, aqueous dispersion and biological contact are closely linked.

The work supports chitosan/alginate nanocomposite gels as a promising platform for oral delivery of poorly soluble flavonoids and provides a statistically explicit framework for future validation. The next stage should test CA-21 in dynamic digestion models containing bile salts and enzymes, mucus-rich intestinal barriers and *in vivo* pharmacokinetic settings. Those experiments would determine whether the improved *in vitro* balance of gastric protection, mucoadhesion and intestinal release translates into higher local or systemic naringenin exposure.

## Author contributions

S. Y. and S. Z. conceived and designed the study. S. Y., J. Z., L. S., and Y. Y. developed the methodology and performed the investigation. S. Y. and Y. Y. curated the data and conducted the formal analysis. J. Z. and L. S. contributed to validation and visualization. S. Y. wrote the original draft. S. Z., Y. Y., L. S., and J. Z. reviewed and edited the manuscript. S. Z. supervised the project, administered the research, and acquired funding. All authors have read and agreed to the published version of the manuscript.

## Conflicts of interest

There are no conflicts to declare.

## Data Availability

The data supporting the findings of this study are available from the corresponding author upon reasonable request.
